# Wave Intensity Analysis in the Human Coronary Circulation in Health and Disease

**DOI:** 10.2174/1573403X10999140226121300

**Published:** 2014-02

**Authors:** Sayan Sen, Ricardo Petraco, Jamil Mayet, Justin Davies

**Affiliations:** International Centre for Circulatory Health, National Heart and Lung Institute, UK

**Keywords:** Collaterals, iFR, wave intensity.

## Abstract

Coronary artery hemodynamics are very different to that of the systemic arteries; unlike the systemic circulation, in the coronary circulation pressure is generated from both the proximal and distal end of the artery – due to the effect of contraction and relaxation of the myocardium on the microvasculature. As a result, the systemic artery hemodynamic model cannot be used to explain the pressure-flow relationship in the coronaries. Wave intensity analysis is an investigative tool that is able to distinguish simultaneous proximal and distal influences on coronary blood flow and is therefore uniquely suitable for the study of coronary haemodynamics. This review discusses the concept behind waveintensity analysis and evaluates how it has been used to characterise and provide new insights on coronary haemodynamics in health and disease.

## INTRODUCTION

**Fig. (1):**
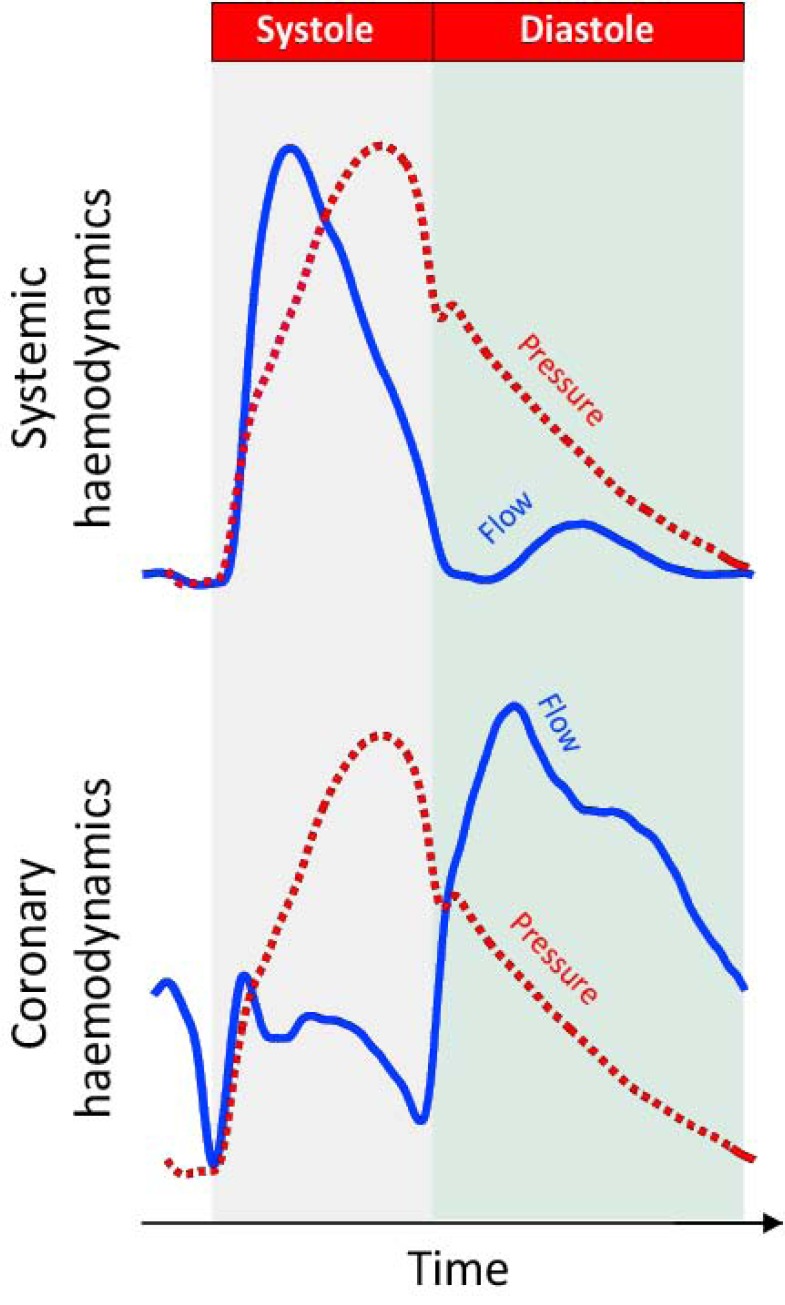
**Difference between the pressure-flow relationship in systemic and coronary arteries.** It can be seen that in a systemic artery changes in pressure and flow closely follow each other. In systole they both increase whilst in diastole they both decrease (upper panel). In the coronary circulation (lower panel) this is not the case. After an initial period in systole when pressure and flow increase together, flow velocity plateaus whilst pressure continues to increase. In early diastole it can be seen that pressure falls rapidly whilst flow velocity increases. Such a paradoxical relationship in early diastole cannot be explained by the traditional haemodynamic model of the systemic vessel.

In all arteries blood flow occurs along a pressure gradient. In systemic arteries this pressure gradient is usually generated at the proximal (aortic) end of the vessel, driving blood towards the capillary bed. The coronary circulation however provides an exception, where fluctuations in pressure not only originate at the proximal end of the vessel, but also originate from the distal (microcirculatory) end of the vessel [1]. These distal-originating pressure changes are actively generated by compression and decompression of the microvasculature, which cause the flow velocity waveform in the coronary artery to be very different from that of a systemic artery such as the aorta (Fig. **1**).

This review describes how it is possible to measure these distal and proximal-originating pressure changes in the coronary arteries using wave intensity analysis, and explores how this tool may give novel insight into how pathological conditions, including valvular heart disease, myocardial and coronary artery disease effect coronary blood flow. 

## WHAT IS WAVE INTENSITY ANALYSIS?

A wave is simply defined as a disturbance that propagates from one place to another with time. Wave intensity analysis can be used to identify and quantify these waves and in its simplest form, net wave intensity, is calculated as the product of the change in pressure (*d*P) and change in flow velocity (*d*U) at a particular point in the cardiac cycle. Its units are of power (Ws^-2^m^-2^), with a negative value indicating a backward-travelling wave and a positive value a forward-travelling wave. In the context of coronary arteries, backward-travelling waves originate from the microcirculatory or distal end of the vessel, and forward-travelling waves from the proximal end of the vessel [2, 3]. Originally used to study gas dynamics, the ability of wave intensity to quantify and separate waves according to their origin and direction of propagation make it an ideal tool to study the coronary circulation; which has pressure perturbations originating from both the proximal and distal ends. 

The effect of the contracting myocardium on blood flow was first demonstrated in an instrumented canine model by Spaan *et al*. [4]. In a series of elegant studies it was demonstrated that as perfusion pressure to the coronary artery was reduced, a critical pressure was reached below which blood flow reversed in a pulsating fashion – indicating a dynamic source of pressure generation from the myocardial bed. In practice the influence of the myocardium on coronary haemodynamics, and therefore waves, is evident by simply inspecting the pressure and flow velocity wave-forms at different times of the cardiac cycle (Fig. **1**). For example during diastole it can be seen that flow velocity increases rapidly whilst intra-coronary pressure falls. Such a combination of falling pressure and increasing blood flow would not fit the haemodynamic model of a systemic vessel which has a single proximal input of pressure. However, it is possible, and indeed normal, in a scenario in which pressure also originates from the distal circulation as occurs in the coronary arteries. The acceleration in blood flow during this period in the face of falling pressure can therefore only be due to a wave, originating from the distal vessel, creating a suction effect which accelerates blood flow into the microcirculation. The origins of this wave have been demonstrated to be secondary to relaxation of the myocardium and decompression of the microvasculature in early diastole [1]; akin to how a compressed sponge sucks up water when suddenly decompressed. Wave-intensity, therefore, identifies and quantifies such waves throughout the cardiac cycle providing a detailed understanding of the haemodynamics of the coronary circulation, by integrating dynamic changes in pressure and flow.

The derivation of waves, estimation of wave speed and techniques for measurements and calculation of net and separated wave-intensity analysis have been extensively described [1-3, 5]. The principle equations for the calculation of coronary artery wave-intensity are highlighted below. The remainder of this review will concentrate on the application of wave intensity analysis to coronary haemodynamics in health and disease, through the use of combined intra-coronary pressure and flow velocity measurements. 

Proximal originating wave intensity:

(1) $$WI_{+}=\frac{1}{4\rho c}{\left(\frac{\mathrm{dP}}{\mathrm{dt}}+\rho c\frac{\mathrm{dU}}{\mathrm{dt}}\right)}^{2}$$

Distal originating wave-intensity:

(2) $$WI_{-}=-\frac{1}{4\rho c}{\left(\frac{\mathrm{dP}}{\mathrm{dt}}+\rho c\frac{\mathrm{dU}}{\mathrm{dt}}\right)}^{2}$$

Net wave-intensity:

(3) $$WI_{\mathrm{net}}=WI_{+}+WI_{-}=\left(\frac{\mathrm{dP}}{\mathrm{dt}}\right)\left(\frac{\mathrm{dU}}{\mathrm{dt}}\right)$$

Where ρ is the density of blood, c wave-speed, dU change in flow velocity and dP change in pressure.

## PRINCIPLE ASSUMPTIONS AND REQUIREMENTS OF WAVE-INTENSITY ANALYSIS

Each wave is a product of changes in pressure and flow velocity at any individual time point in the cardiac cycle (Equation 1-3). It is therefore important to ensure high quality pressure and flow velocity envelopes are obtained when measurements are taken to ensure the subsequent derivation of wave-intensity is an accurate reflection of underlying phasic coronary hemodynamics. Whilst achieving a high fidelity flow wave envelope can be time consuming it is usually obtainable by operators experienced in flow velocity measurements and is aided by the use of a combined pressure and Doppler tipped wires that enable simultaneous capture of pressure and flow velocity. 

The main assumption in the derivation of wave intensity itself lies with the use of the single point measure of local wave-speed [5]. An initial pre-requisite for the use of wave-intensity in the coronary circulation was the development of a new means of estimating wave-speed when making measurements of pressure and flow velocity at only a single point in the coronary artery. Using the relationship between simultaneous pressure and velocity and the density of blood as a constant, a new equation was derived to estimate local wave speed (Equation 4). Whilst initial validation work involved assessment of this in the aorta it is applicable in any artery in which simultaneous measure of pressure and velocity can be made, and is correct when taken over at least one complete cardiac cycle. 

Equation for single point estimate for wave-speed (c):

(4) $$c=\frac{1}{\rho }\sqrt{\frac{\sum dP^{2}}{\sum dU^{2}}}$$

## MECHANICS OF WAVES IN NORMAL CORONARIES

**Fig. (2):**
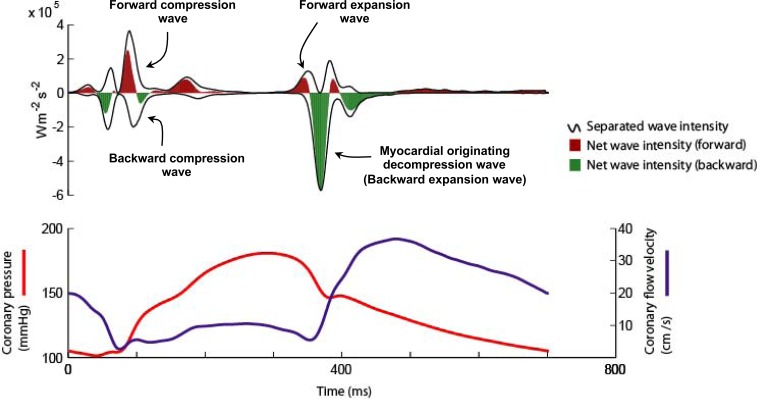
**Coronary wave intensity analysis and intra-coronary pressure and flow velocity relationship.** (Upper panel): Waves originate from both the proximal (positive values) and distal circulation (negative values). Waves from both ends of the arteries are present in both systole and diastole. The 4 predominant waves of the cardiac cycle are highlighted. The predominant determinant of coronary flow is the myocardial originating decompression wave (or backward expansion wave), which has a suction-like effect. (Lower Panel): Wave intensity can be used to explain the pressure-flow relationship in the coronary artery. For example it can be seen that the paradoxical fall in pressure and yet increase in flow velocity during early diastole is actually due to the myocardial originating decompression wave sucking blood into the coronary. This wave originates in the distal circulation due to relaxation of the myocardium and decompression of the microvasculature.

Six waves have been identified within the cardiac cycle [1]. These can be classified with respect to their origin (proximal, forward-travelling waves or distal, backward-travelling waves) and according to their effect on blood flow (expansion / suction waves or compression /pushing waves) [1]. Two proximal and two distal originating waves predominate within the cardiac cycle (Fig. **2**). At the start of the cardiac cycle and the onset of myocardial contraction it can be seen that there are two waves occurring almost simultaneously, one from the proximal end and one from the distal end of the vessel. The wave from the proximal end is due to pressure originating from aorta following left ventricular ejection; this forward compression wave accelerates flow. The wave from the distal vessel is secondary to myocardial contraction and compression of the microvasculature. This generates a backward-travelling compression wave that decelerates flow. During systole it can be seen that these opposing waves result in a plateau of coronary blood flow despite intra-coronary pressure continuing to increase. At the onset of diastole and relaxation of the myocardium two further waves are generated. During this early period of diastole the wave originating from the proximal (aortic) end of the artery generates a forward-travelling suction effect, decelerating blood flow. This coincides with the generation of a wave from the distal vessel which is secondary to decompression of the microvasculature during myocardial relaxation. The effect of this wave is to accelerate blood flow. Once again the two waves oppose each other, however, the dominance of the myocardial originating decompression wave results in net acceleration of blood flow; thereby creating the phenomenon of increasing flow velocity with falling intra-coronary pressure. 

The ability of wave-intensity to characterise the interaction between the myocardium and coronary blood flow at each point within the cardiac cycle provide novel insights into conditions that influence the interplay between the myocardium and coronary artery.

## WAVE INTENSITY AND MYOCARDIAL – CORONARY INTERACTION IN LEFT VENTRICULAR HYPERTROPHY

Left ventricular hypertrophy in the context of hypertension is associated with negative remodelling of the myocardium involving processes such as myocardial fibrosis and perivascular necrosis [6]. These changes have been demonstrated to impair diastolic function and often present clinically with symptoms of exertional dyspnoea. 

In a study using wave intensity analysis to compare the myocardial coronary interaction in normal patients and patients with left ventricular hypertrophy it was demonstrated that these myocardial changes had a significant influence on coronary haemodynamics [1]. The myocardial originating decompression wave, the predominant accelerant of coronary blood flow was significantly reduced in patients with left ventricular hypertrophy. The aetiology of this is likely multi-factorial with contributions from negative remodelling of the myocardium and microvasculature reducing basal microvascular volume, and simultaneously limiting the ability of the myocardium to relax during diastole (diastolic dysfunction); thereby reducing the volume of blood drawn into the microcirculation during diastole. The net effect is to detrimentally alter coronary haemodynamics and thereby feasibly contribute to further negative remodelling. This perfusion myocardial mass mismatch could provide some of the physiological basis of the clinical manifestation of left ventricular hypertrophy, namely exertional dyspnoea and angina in the context of unobstructed coronaries. 

## WAVE-INTENSITY ANALYSIS AND MYOCARDIAL CORONARY INTERACTION DURING BIVENTRICULAR PACING

Cardiac re-synchronisation in patients with left bundle branch block and left ventricular ejection fraction of less than 35% has been shown to reduce morbidity and mortality [7]. However the effect of myocardial resynchronisation and varying atrio-ventricular (AV) delay delay on coronary blood flow has not been extensively studied. Kyriacou and colleagues used wave- intensity analysis to study the coronary haemodynamic effect of biventricular pacing and myocardial resynchronisation [8]. In this study wave intensity was used to help differentiate the effect of ventricular resynchronisation from that of varying AV delay (and therefore pre-load) on myocardial contraction, myocardial relaxation and coronary blood flow. The results showed that improvements in biventricular pacing significantly improved both myocardial contraction and relaxation leading to a significant increase in the myocardial decompression wave and therefore blood flow. However, it was also demonstrated that the beneficial effect of resynchronisation on the myocardium, and therefore coronary blood flow, could be significantly altered and even negated by simply selecting a sub-optimal AV delay (thereby impairing pre-load). As a result at the same heart rate but different AV delays there were significant changes in the myocardial-originating decompression wave and coronary blood flow; the highest flows were achieved at the optimal AV delay. By differentiating the effect of ventricular synchronisation and AV optimisation on coronary haemodynamics this study provides further evidence of the importance of AV optimisation in patients with cardiac resynchronisation devices. 

## WAVE INTENSITY AND MYOCARDIAL – CORONARY INTERACTION IN AORTIC STENOSIS

As the predominant determinant of flow, the behaviour of the myocardial-originating decompression wave was assessed in patients with severe aortic valve stenosis using the Trans-catheter Aortic Valve Replacement (TAVR) model [9]. This model of aortic valve stenosis circumvents many of the limitations of prior *in vivo* work, and provides a unique opportunity to study the dynamic effect of left ventricular after load on coronary haemodynamics immediately before and after aortic valve replacement. 

**Fig. (3):**
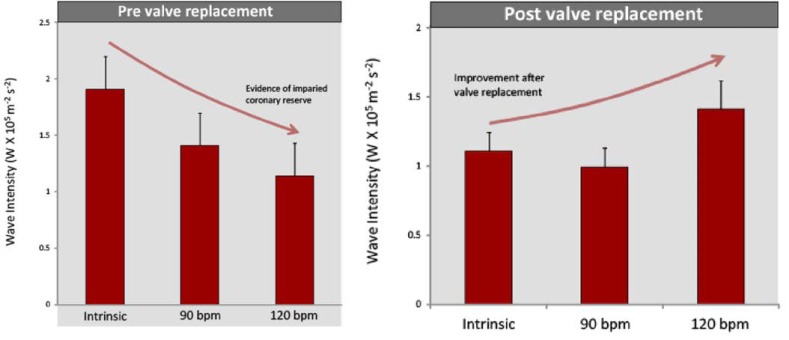
**Coronary wave-intensity and severe aortic stenosis. **Coronary physiological reserve varied considerably pre and post TAVR. Physiological reserve was assessed by measuring the microcirculatory decompression (suction) wave at rest and then by pacing at 90 and 120 bpm. Before TAVR, the myocardial decompression (suction) wave decreased with increasing heart rate. After TAVR, the reverse was observed, and the myocardial decompression (suction) wave increased with increasing heart rate.

In a study of 11 patients undergoing TAVR wave intensity analysis was performed on simultaneous intra coronary pressure and flow velocity measurements made in the left main stem prior to and immediately after valve insertion, at three different heart rates (varied via right ventricular pacing). Prior to insertion of the aortic valve, wave-intensity analysis demonstrated that there was a significant difference in the myocardial-originating decompression wave with increasing heart rate. As the heart was paced at increasing rates, the magnitude of the myocardial-originating decompression wave progressively decreased (Fig. **3**, Upper Panel). This is the opposite response to that normally expected with increased heart rate [10] and suggests that as cardiac work increases, in these patients with severe aortic stenosis, the ability of the myocardium to increase blood flow paradoxically decreases. 

**Fig. (4):**
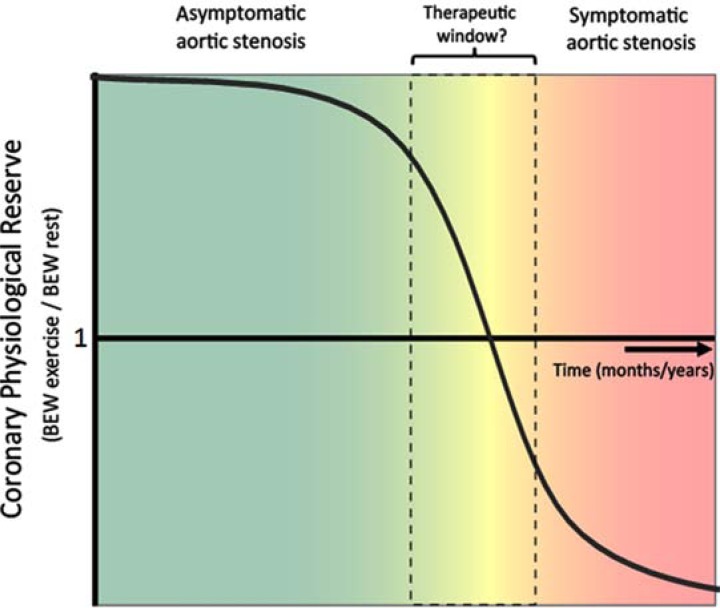
**Schematic of behaviour of coronary physiological reserve with progression of aortic stenosis over time. **At present surgery is advocated in aortic stenosis in patients that have symptoms at which point prognosis is very poor. However, wave intensity analysis suggests that as aortic stenosis progresses, the ability of the myocardial expansion wave to increase will eventually progressively diminish. The decrease in this wave could potentially provide a tool to more accurately monitor the physiological impact and therefore severity of aortic stenosis. A more physiological approach may help identify patients that require valve replacement earlier when they are more likely to be a better substrate for surgical/ percutaneous intervention.

Post valve insertion the effect of pacing on coronary haemodynamics was reassessed. This showed, that the effects of increasing heart rate were reversed: the magnitude of the myocardial-originating decompression wave progressively increased with increasing heart rate, indicating a restoration of ‘coronary physiological reserve’ (Fig. **3**, lower panel). These contrasting haemodynamic responses to increased work load pre and post aortic valve replacement; isolate the haemodynamic effect of aortic stenosis over and above the effect of LVH in this patient group. Moreover, these findings provide further insight into the mechanisms responsible for the symptoms, such as angina and exertional dyspnoea in patients with severe AS; namely a progressive reduction in myocardial reserve and therefore inability to increase blood flow to meet increased work load. The progressive reduction of coronary physiological reserve, with worsening aortic stenosis, may serve as a potential new measure of aortic stenosis significance (Fig. **4**); especially as it becomes increasingly feasible to perform wave-intensity using non invasive techniques. 

## WAVE INTENSITY AND MYOCARDIAL – CORONARY INTERACTION IN CORONARY STENOSIS ASSESSMENT

**Fig. (5):**
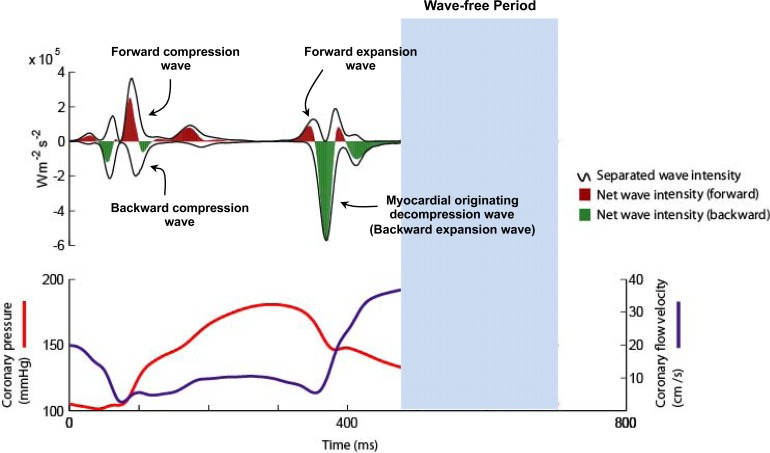
**The diastolic wave-free period – a window in the cardiac cycle free of accelerative and decelerative forces. **A period in diastole free of accelerative and decelerative forces has been demonstrated to be the most appropriate time in the cardiac cycle to assess the fluid dynamics of an upstream coronary stenosis. Whilst previously identified using manual analysis of pressure and flow, wave intensity permits identification of such a period (termed the diastolic wave-free period) using automated algorithms. This period has been demonstrated to be associated with a more stable and lower microvascular resistance than the rest of the cardiac cycle. The instant wave-free ratio (iFR) is a pressure only index of stenosis severity that is calculated over this window.

Over 30 years ago Gould used combined pressure and flow velocity measurements to determine the most appropriate period within the cardiac cycle to assess a coronary stenosis [11]. In a series of studies he demonstrated that stenosis assessment was clearly confounded by the influence of the contracting and relaxing myocardium on blood flow. Through meticulous manual post hoc analysis of haemodynamic data he identified a period free of these ‘accelerative and decelerative forces’ of the myocardium that confound assessment of stenosis severity. Recently, wave intensity analysis has been applied to the assessment of coronary stenoses [12]. The ability of wave intensity analysis to identify the beginning and end of these ‘accelerative and decelerative forces’ make it an ideal tool to identify a period in the cardiac cycle when such forces are absent. This has resulted in the isolation of a wave-free period in diastole, during which there are no accelerative and decelerative forces (Fig. **5**, upper panel). During this period of the cardiac cycle intra-coronary pressure and flow decline together and microvascular resistance is more stable and lower than that over the rest of the cardiac cycle; making it suitable for a resting pressure-only assessment of the haemodynamic significance of coronary stenoses. The instantaneous wave-free ratio (iFR) is a vasodilator free pressure derived index of stenosis severity measured during this wave-free window; it has been demonstrated to have good agreement with existing pressure derived indices [12-16]. Whilst further work is necessary to achieve a better understanding of the clinical utility of iFR and its limitations, it demonstrates the potential of highly technical methods such as wave-intensity to provide new clinically relevant investigational tools for identification of abnormalities within the coronary circulation. 

## WAVE INTENSITY AND COLLATERAL BLOOD FLOW 

The impact of coronary collateral circulation on mortality was characterised in a meta-analysis which demonstrated a 36% reduction in mortality in patients with evidence of collaterals [17, 18]. Both intra-coronary pressure and flow velocity have been used to identify and quantify the effect of collateral blood flow [19-21]. The most established index for the intra-coronary assessment of collaterals is the Collateral Flow Index [22]. This uses intracoronary occlusive pressure- or velocity and expresses collateral flow as a fraction of flow during vessel patency [21]. In contrast the investigation of the effect of coronary collaterals using wave-intensity remains in its infancy. 

In a recent study in patients post acute coronary syndrome the magnitude of the myocardial originating decompression wave was found to correlate closely to the mass of viable myocardium subtended [23]; suggesting that this wave in the native coronary artery could vary in other conditions where the amount of myocardium can change, as occurs in collateral circulations. Whilst further investigation is required in this area the agreement of wave-intensity derived iFR to fractional flow reserve in multi vessel disease also supports the possibility that that wave-intensity could be a useful tool in characterising native coronary collateral flow [12]. 

## CONCLUSION

The unique haemodynamics of coronary blood flow (with pressure and flow perturbations at both the distal and proximal end of the coronary artery) means that its detailed characterisation by measuring pressure or flow in isolation is limited. The integration of both these variables using wave-intensity analysis allows a more comprehensive assessment of coronary haemodynamics. This has permitted the use of this novel investigational tool to elucidate potential mechanisms of symptom aetiology in patients with LVH, AS and coronary stenoses. The ability to temporally distinguish the predominant determinants of blood flow at specific points in the cardiac cycle suggests that wave-intensity has the potential for detailed analysis of the haemodynamic effect of collaterals. More work is required to elucidate this potential.
